# Identification of a putative nuclear localization signal in the tumor suppressor maspin sheds light on its nuclear import regulation

**DOI:** 10.1002/2211-5463.12626

**Published:** 2019-05-29

**Authors:** Jeffrey Reina, Lixin Zhou, Marcos R. M. Fontes, Nelly Panté, Nathalie Cella

**Affiliations:** ^1^ Department of Cell and Developmental Biology Institute of Biomedical Science of University of São Paulo Brazil; ^2^ Department of Zoology University of British Columbia Vancouver BC Canada; ^3^ Department of Physics and Biophysics Institute of Biosciences São Paulo State University (UNESP) Botucatu Brazil

**Keywords:** import assay, mammary serine protease inhibitor, maspin, nuclear localization signal, nuclear transport, tumor suppressor

## Abstract

The tumor suppressor activity of maspin (mammary serine protease inhibitor) has been associated with its nuclear localization. In this study we explore the regulation of maspin nuclear translocation. An *in vitro* nuclear import assay suggested that maspin can passively enter the nucleus. However, *in silico* analysis identified a putative maspin nuclear localization signal (NLS), which was able to mediate the nuclear translocation of a chimeric protein containing this NLS fused to five green fluorescent protein molecules in tandem (5GFP). Dominant‐negative Ran‐GTPase mutants RanQ69L or RanT24N suppressed this process. Unexpectedly, the full‐length maspin fused to 5GFP failed to enter the nucleus. As maspin's putative NLS is partially hidden in its three‐dimensional structure, we suggest that maspin nuclear transport could be conformationally regulated. Our results suggest that maspin nuclear translocation involves both passive and active mechanisms.

**Abbreviations**
5GFP: five green fluorescent proteins
DAPI: 4′,6‐diamidino‐2‐phenylindole
EGF: epidermal growth factor
Kabβ: karyopherin‐β
maspinFL: full‐length maspin
maspin: mammary serine protease inhibitor
NLS: nuclear localization signal
RRL: rabbit reticulocyte lysates
WT: wild‐type

Maspin (mammary serine protease inhibitor), also known as SerpinB5, is a potential tumor suppressor first identified in breast tissue 1. It is now well established that maspin is expressed by most epithelia 2 and has diverse biological activities, including inhibition of tumor growth and invasion, and regulation of cell adhesion, migration, apoptosis, gene transcription and oxidative stress response 3. Maspin tumor suppressor activity is complex and appears to be cell‐type and tissue‐context dependent. Downregulation of maspin expression has been observed in some tumor types 1, 4, 5, whereas others show an opposite trend 6, 7, 8, 9, 10. Interestingly, increasing evidence indicates that maspin nuclear localization, rather than its level of expression, correlates with good prognosis and tumor suppression 10, 11, 12, 13, 14. Evidence indicates that nuclear maspin promotes tumor suppression by regulating gene transcription. It inhibits histone deacetylase I 15 and it interacts with the promoter of the macrophage colony‐stimulating factor‐1 (*CSF‐1*) and the estrogen‐related receptor α (*ESRRA*) genes 13, which are both implicated in tumor progression 16, 17. In addition, nuclear maspin inversely correlates with the proliferative state in invasive ductal breast cancer and breast cancer cell lines 18. These data underscore the importance of understanding the molecular mechanism underlying maspin nucleocytoplasmic traffic, which can lead to new cancer therapeutic targets. Regulated nuclear transport is a signal‐mediated process dependent on nuclear transporters of the karyopherin‐β (Kabβ) superfamily, also called importins and exportins 19. The 20 different human Kabβ subfamilies recognize a nuclear localization signal (NLS) within the cargo polypeptide. The cargo–importin complex binds to nucleoporins of the nuclear pore complex and to the small GTPase Ran, which provides both energy and directionality to the transport. As a 42 kDa protein, maspin can potentially enter the nucleus by passive diffusion 20. However, when maspin cDNA was transfected into mouse mammary tumor TM40D cells, maspin was found exclusively in the cytoplasm 21. In addition, we found that a fraction of cellular maspin accumulates in the nucleus in epidermal growth factor (EGF) ‐treated MCF‐10A cells and it is predominantly cytoplasmic in the mouse mammary gland 22. Based on the observations described above, we hypothesized both passive and active/regulated mechanisms regulate maspin nucleocytoplasmic shuttling. To test this hypothesis, we reconstituted maspin nuclear import *in vitro* using digitonin‐permeabilized HeLa cells. We observed that maspin promptly translocates to the nucleus in the absence of exogenous cytosol or energy‐regenerating solution, indicating that maspin enters the nucleus passively. Using the nuclear localization signal predictor cnls mapper 23, we identified a putative bipartite NLS of 28 amino acids in the maspin protein sequence. In order to investigate if this sequence plays a role on active/regulated maspin nuclear import, full length maspin and the maspin putative NLS sequence were cloned into a plasmid encoding five green fluorescent protein molecules in tandem (5GFP), generating maspinFL–5GFP and 5GFP–maspinNLS constructs, respectively. When the corresponding proteins are expressed, it is expected that they do not passively diffuse because 5GFP is too large to passively translocate to the nucleus 20. Surprisingly, maspin NLS, but not maspin full length, was able to drive nuclear import of the 5GFP construct, indicating that this peptide sequence can mediate an active transport to the nucleus. As active nuclear transport requires energy provided by Ran‐GTPase‐mediated GTP hydrolysis, we further investigate 5GFP–maspinNLS nuclear transport in the presence of the RanQ69L and RanT24N mutants, which are deficient in GTP hydrolysis or do not bind to GTP, respectively, and therefore act as dominant negative inhibitors of signal‐ and energy‐dependent nuclear transport 24, 25. We observed that 5GFP–maspinNLS nuclear import was completely inhibited when Ran‐GTPase mutant plasmids were co‐transfected in HeLa cells. Herein, we provide evidence that maspin translocates to the nucleus passively. In addition, we identified a buried NLS which is necessary and sufficient for nuclear import of a 5GFP construct in a Ran‐GTPase‐dependent manner. This NLS was, however, unable to drive nuclear translocation of full length maspin in the tested conditions.

## Materials and methods

### Cell culture

HeLa cells were obtained from the American Type Culture Collection and were cultured in Dulbecco's modified Eagle's medium (Sigma‐Aldrich, Sigma‐Aldrich Canada Co., Oakville, Ontario, Canada) supplemented with 5% fetal bovine serum (Sigma‐Aldrich), 1% penicillin–streptomycin, 1% l‐glutamine (Cellgro, Manassas, VA, USA) and 1% sodium pyruvate (Thermo Fisher Scientific, Waltham, MA, USA). Cells were maintained at 37 °C with 5% CO_2_.

### Nuclear import assay in digitonin‐permeabilized cells

BSA covalently attached to the NLS of SV40T antigen (CGGGPKKKRKVED) at a ratio of 5 : 1 (NLS:BSA) was custom made (Sigma‐Genosys, Spring, Texas, USA). Cy3 protein labeling was done with the Cy3 bis‐Reactive Dye Pack (GE Healthcare Amersham, Little Chalfont, Buckinghamshire, UK) following the manufacturer's instructions.

HeLa cells were grown on glass coverslips until they were 40–60% confluent, washed once with phosphate buffered saline (PBS) and once with import buffer (20 mm HEPES pH 7.4, 110 mm potassium acetate, 1 mm EGTA, 5 mm sodium acetate, 2 mm magnesium acetate, 2 mm DTT and 10 μg·mL^−1^ protease inhibitors). For permeabilization, cells were incubated with digitonin (20 μg·mL^−1^) for 3 min at room temperature and washed three times with import buffer. Permeabilized cells were incubated with or without an energy regenerating system (0.4 mm ATP, 0.45 mm GTP, 4.5 mm phosphocreatine, 18 U·mL^−1^ phosphocreatine kinase, 1.6 mg·mL^−1^ BSA) and 20% cytosol extract obtained from nuclease‐treated rabbit reticulocyte lysate (RRL) (Promega, Madison, WI, USA) in the presence of 0.2 μg of 70 kDa fluorescent Dextran Texas Red (Thermo Fisher Scientific), 2 μg of Cy3‐labeled BSA fused to SV40 NLS sequence (Sigma‐Genosys), or Cy3‐labeled human recombinant maspin (Peprotech, Rocky Hill, NJ, USA) for 30 min at 37 °C. Next, the cells were washed three times with import buffer and fixed with 3% paraformaldehyde for 10 min. Finally, the cells were washed three times for 5 min with PBS and mounted onto microscope slides using ProLong Diamond Antifade Mountant with 4′,6‐diamidino‐2‐phenylindole (DAPI) (Thermo Fisher Scientific). Samples were visualized using a Fluoview FV1000 confocal laser scanning microscope (Olympus, Quebec, Canada).

### Maspin NLS prediction

cnls mapper 23 was used to predict a putative NLS using human maspin amino acid sequence (UniProt identifier: P36952‐1) and cut‐off score of 6.0.

### Plasmids

To generate the 5GFP–maspinNLS construct, two synthetic primers (5′‐GATCCAAACTAATCAAGCGGCTCTACGTAGACAAATCTCTGAATCTTTCTACAGAGTTCATCAGCTCTACGAAGAGACCCTATGCAG‐3′ and 5′‐GATCCTGCATAGGGTCTCTTCGTAGAGCTGATGAACTCTGTAGAAAGATTCAGAGATTTGTCTACGTAGAGCCGCTTGATTAGTTTG‐3′) were designed to include a putative bipartite NLS region in human maspin. The synthetic DNA for maspinNLS containing adapters of the BamHI restriction enzyme at each end were annealed, and the annealed DNA fragments were ligated to the BamHI site at the C‐terminal coding sequence of 5GFP. The construct was confirmed by DNA sequencing.

To generate maspinFL–5GFP plasmid, human maspin cDNA was first subcloned from SERPINB5 plasmid (Origene, Catalogue number: RC224287) to pEGFP‐C3 vector. The resulting maspin–GFP plasmid was used in a PCR reaction using Mas‐NheI‐F2 (forward) and Mas‐Nhe‐R‐1 (reverse) primers: 5′‐GAA CCG TCA GAT CCG CTA GCG CT‐3′ and 5′‐CAC TAG CTA GCG GAG AAC AGA ATT TGC CAA AGA AAA T‐3′. The NheI restricted maspin PCR product was inserted into the NheI‐digested pEGFP‐GFP5 vector and ligated with T4 DNA ligase (New England Biolabs, Ipswich, MA). The ligation products were transformed by heat shock into Subcloning Efficiency™ DH5α™ Competent Cells (Thermo Fisher Scientific) and colonies were screened for the presence of the insert. Those positive for the insert were confirmed by sequencing.

The pcDNA‐RanWT‐mRFP1‐polyA (Addgene plasmid no. 59750), pTK21 (RanT24N) (Addgene plasmid no. 37396) and pmCherry‐C1‐RanQ69L (Addgene plasmid no. 30309) were gifts from Yi Zhang 26, Ian Cheeseman 27 and Jay Brenman 28, respectively.

### Transfection and co‐transfection of plasmids

HeLa cells were grown on glass coverslips in a 24‐well dish without antibiotics. DNA of 250 ng of each plasmid was transfected with Lipofectamine 2000 (Invitrogen). Twenty‐four hours post‐transfection cells were fixed with 3% paraformaldehyde in PBS pH 7.4 for 10 min, washed three times with PBS and mounted onto microscope slides using ProLong Diamond Antifade Mountant with DAPI (Invitrogen). Cells were visualized with a Fluoview FV1000 confocal laser scanning microscope (Olympus).

### Quantification of nuclear import

Quantification was as described in 29. Briefly, the fluorescence intensity of defined areas (20 × 20 pixels) was measured in the nucleus (*F*_n_) and in the cytoplasm (*F*_c_) using ImageJ (National Institutes of Health, Bethesda, MD, USA). The mean background intensity (MB) was also measured. The ratio of nucleus to cytoplasm fluorescence intensity was calculated using the equation: *F*_n_/*F*_c_ = (*F*_n_ − MB)/(*F*_c_ − MB). Data were obtained from at least 50 cells per experiment. Normal distribution was tested for each set of data using the Shapiro–Wilk normality test. In the case of normal distribution, one‐way ANOVA was used to compare means between groups. In the case of non‐normal distribution, a Kruskal–Wallis test was used instead. To compare all groups Dunn's *post‐hoc* test was used. All the statistical analyses were performed using prism (GraphPad Software Inc., La Jolla, CA, USA) and a *P*‐value < 0.05 was considered significant. All data are represented as mean ± 95% confidence interval.

## Results

### Maspin diffuses into the nucleus of digitonin‐permeabilized cells

**Figure 1:**
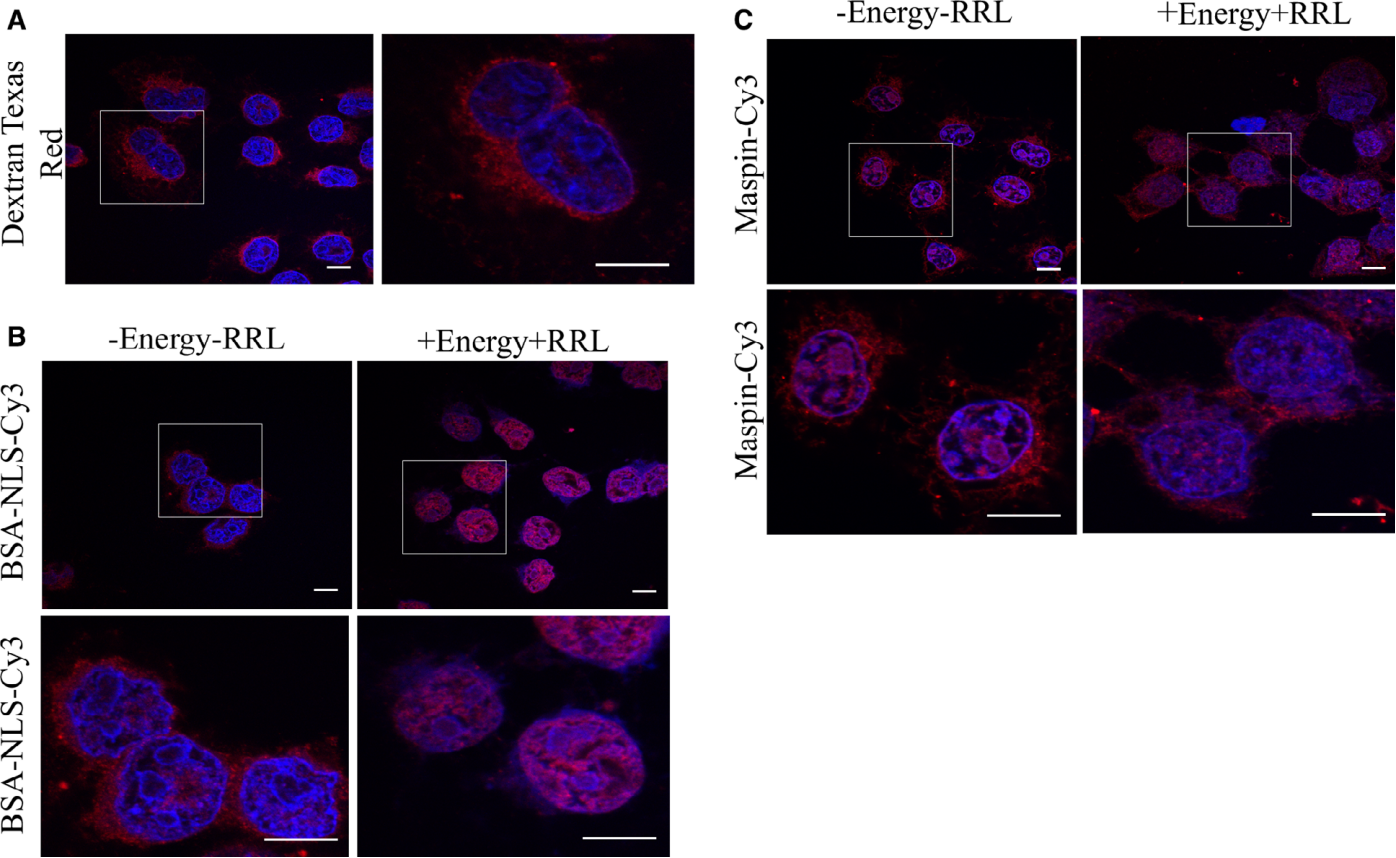
Maspin diffuses into the nucleus of digitonin‐permeabilized HeLa cells. (A) HeLa cells were incubated with 70 kDa Dextran Texas Red to confirm that the nuclear membrane was intact after digitonin permeabilization. (B,C) Digitonin‐permeabilized HeLa cells were incubated with BSA–NLS–Cy3 (B) or maspin–Cy3 (C) in the presence or absence of cytosol extract (+RRL and −RRL, respectively) and with or without an energy‐regenerating system (+Energy, −Energy, respectively). DAPI was used to stain the nuclei. Scale bars: 10 μm.

Maspin is found in the nucleus, cytoplasm or both compartments depending on the physiological conditions and cell‐type 30. As a 42 kDa protein, maspin may be able to translocate passively to the nucleus. In order to test this, we took advantage of the widely used nuclear import assay, where the plasma membrane is selectively permeabilized by digitonin, releasing cytoplasmic components and leaving the nuclear envelope intact 31. HeLa cells were permeabilized with digitonin and the integrity of the nuclear envelope was tested with Texas Red‐labeled 70 kDa dextran. In this assay fluorescence was detected in the cytoplasm only (Fig. 1A), confirming the integrity of the nuclear envelope. Recombinant maspin or BSA covalently linked to the NLS of SV40 T antigen was conjugated to Cy3 and assayed in digitonin‐permeabilized HeLa cells in the presence or absence of an energy regenerating system (+ energy or − energy) and RRL, a source of cytoplasmic factors. As expected, BSA–NLS–Cy3 was promptly detected in the nuclei of digitonin‐permeabilized cells in the presence of energy and RRL (Fig. 1B, right hand panels), but not in the absence of them (Fig. 1B, left hand panels). Interestingly, maspin–Cy3 entered the nucleus irrespective of the presence of energy and RRL (Fig. 1C). This result indicates that maspin can potentially enter the nucleus passively.

### Maspin amino acids 87–114 drive nuclear translocation of the chimeric protein 5GFP–maspinNLS

**Figure 2:**
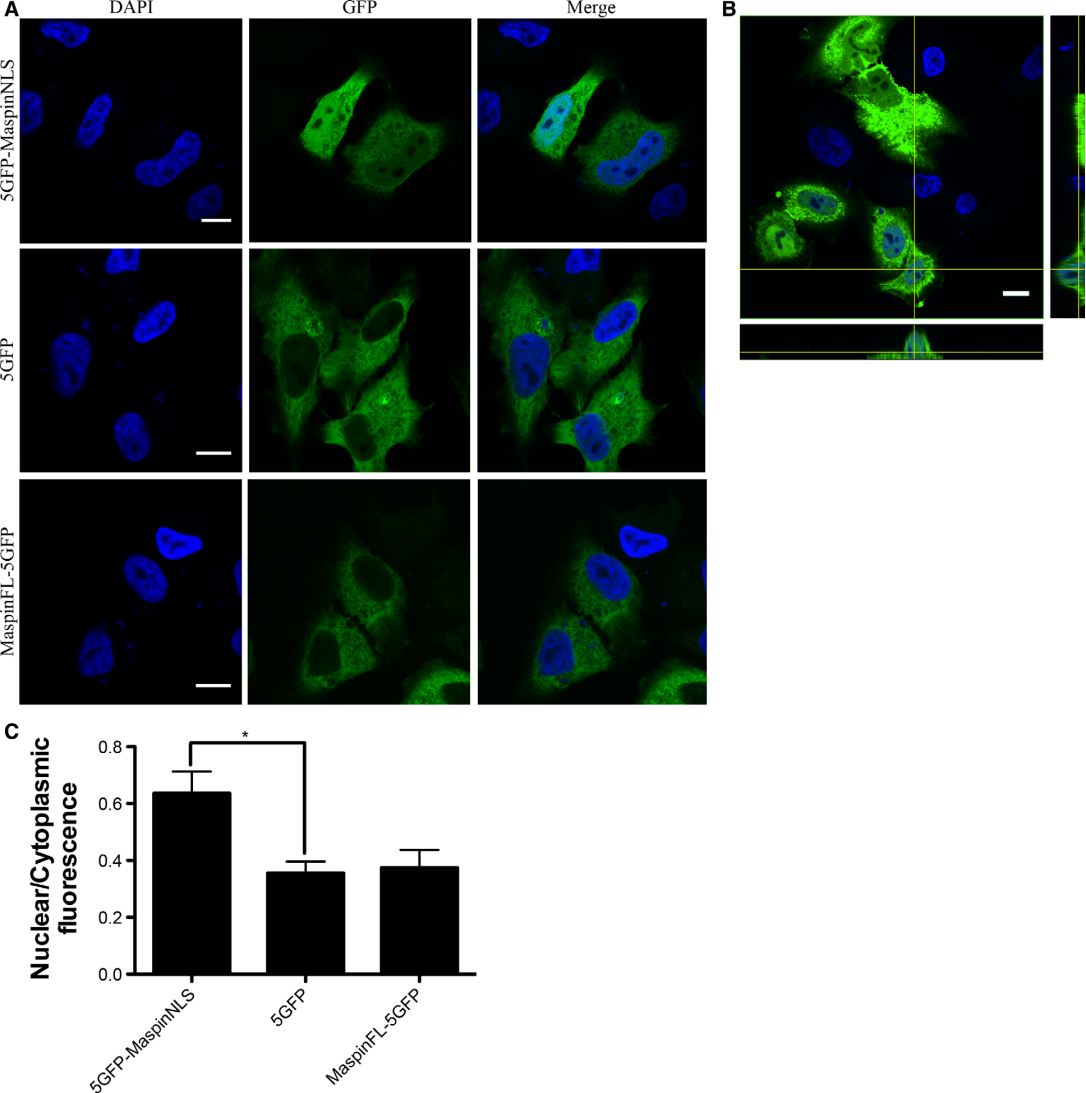
Maspin putative NLS, but not maspinFL, fused to 5GFP induces nuclear transport of the chimera protein. (A) HeLa cells were transfected with 5GFP–maspinNLS, maspinFL–5GFP and 5GFP plasmids. (B) *XZ* and *YZ* projections of HeLa cells transfected with 5GFP–maspinNLS. After 24 h cells were fixed and visualized with a confocal microscope. DAPI was used to stain the nuclei. Scale bars: 10 μm. (C) Quantification of nuclear to cytoplasmic fluorescence ratio from confocal microscope images. Bars show mean ± 95% confidence interval. *P < 0.05, Kruskal–Wallis test followed by Dunn's test, *n* ≥ 50 cells, *N* = 4 independent experiments.

The observation that maspin can diffuse into the nucleus does not exclude the possibility that a regulated mechanism takes place. We have previously reported the existence of different maspin isoforms in the cell 22, 32. Maspin is found in different cellular compartments other than the nucleus, including mitochondria 33, endoplasmic reticulum‐associated vesicles 2, plasma membrane 34 and exosomes 35. Therefore, maspin isoforms may be located in different compartments, implying a diverse regulation of subcellular localization. In addition, maspin's nucleocytoplasmic distribution is a determinant of its biological function as a tumor suppressor and correlates with tumor prognosis. These observations underscore the importance and complexity of the intracellular traffic control of maspin. To determine whether maspin enters the nucleus by a carrier‐dependent mechanism that requires the presence of at least an NLS on maspin, we took advantage of the cnls mapper algorithm 23. The predicted maspin NLS sequence is the 28 amino acid sequence KLIKRLYVDKSLNLSTEFISSTKRPYAK, which was called maspinNLS. In order to distinguish between passive and regulated nuclear translocation, maspinNLS or full‐length maspin (maspinFL) was fused to five green fluorescent protein molecules in tandem (5GFP), generating proteins that are above the diffusion limit of the nuclear pore complex 20. 5GFP–maspinNLS and maspinFL–5GFP constructs were transfected in HeLa cells. As a control, HeLa cells were also transfected with the 5GFP plasmid. The subcellular localization of the resulting chimeric proteins was assessed 24 h post‐transfection using confocal laser scanning microscopy. As expected, without NLS, 5GFP was localized in the cytoplasm of the transfected cells (Fig. 2A, middle panels). However, maspinNLS was able to drive nuclear transport of the 5GFP chimera protein (Fig. 2A, upper panels), which we confirmed to be inside the nuclei by z‐stack tridimensional reconstruction (Fig. 2B). This suggests that maspinNLS could potentially be responsible for maspin's nuclear translocation in the native molecule (Fig. 2A, upper panels). Unexpectedly, maspinFL–5GFP was not detected in the nucleus (Fig. 2A, lower panels). Quantification of the nuclear to cytoplasmic fluorescence ratio (*F*_n_/*F*_c_) in these cells showed that cellular localization of maspinFL–5GFP was indistinguishable from 5GFP‐transfected cells (Fig. 2C). The same result was observed in transfected MCF‐10A cells (data not shown), a non‐transformed mammary epithelial cell line, indicating that the exclusion of maspinFL–5GFP from the nucleus is not restricted to the HeLa cell line.

### Nuclear import of 5GFP–maspinNLS depends on Ran‐GTPase

**Figure 3:**
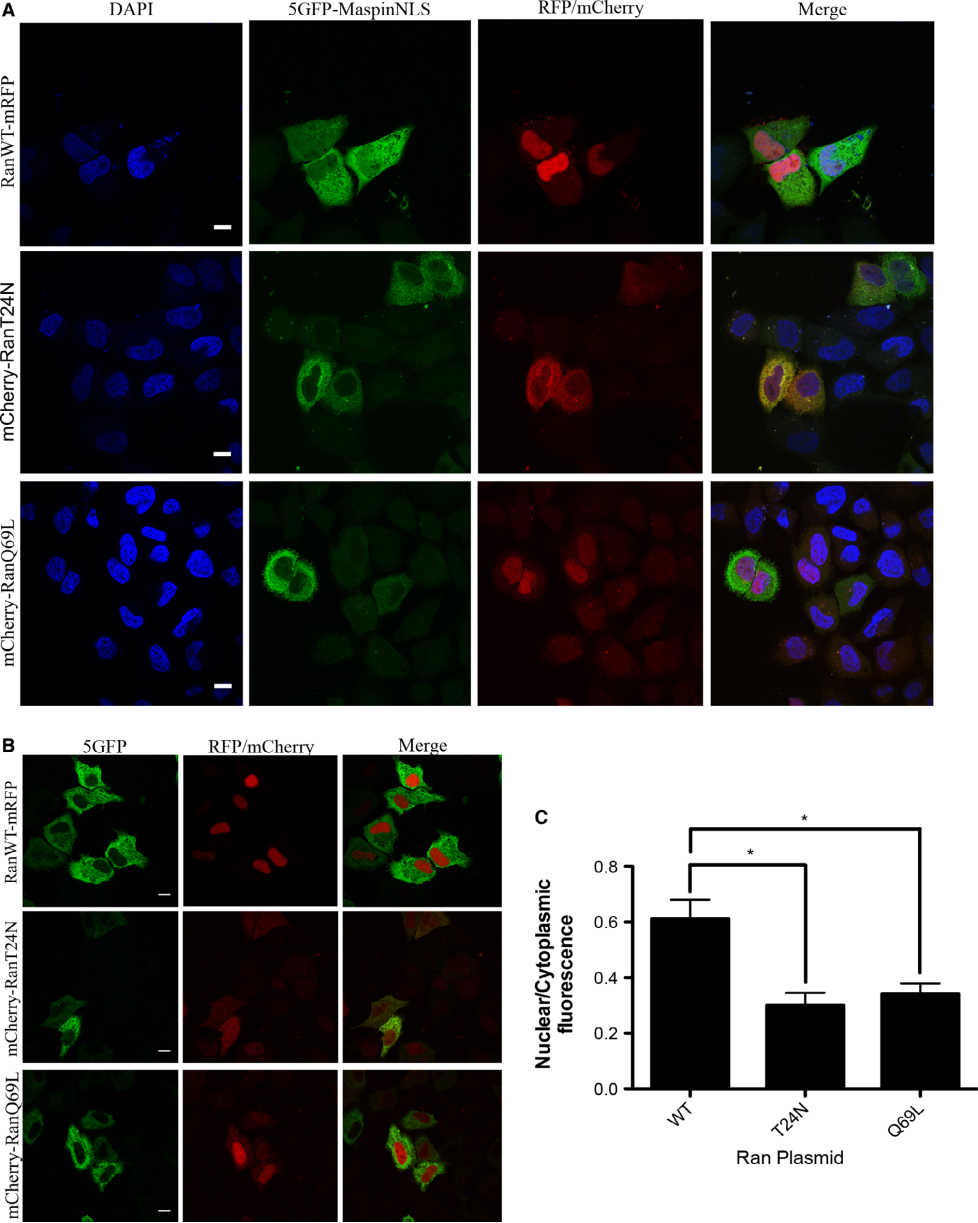
Ran mutants inhibited 5GFP–maspinNLS nuclear import in HeLa cells. (A,B) HeLa cells were co‐transfected with plasmids 5GFP–maspinNLS (A) or 5GFP (B) plasmids and RanWT–mRFP, mCherry–RanT24N, or mCherry–RanQ69L plasmids. After 24 h cells were fixed and visualized with a confocal microscope. DAPI was used to stain the nuclei. Scale bars: 10 μm. (C) Quantification of nuclear to cytoplasmic fluorescence ratio from confocal microscope images. Graph bars show mean ± 95% confidence interval. *P < 0.05, Kruskal–Wallis test followed by Dunn's test, *n* ≥ 50 mCherry‐positive cells, *N* = 4 independent experiments.

Active nuclear transport depends on the Ran‐GTPase, although non‐conventional mechanisms, which are both importin and Ran‐GTP independent, do occur 36. To further characterize the mechanism of maspin nuclear translocation, HeLa cells were cotransfected with 5GFP–maspinNLS together with plasmids encoding fluorescent wild‐type (WT) Ran‐GTPase, RanQ69L or RanT24N mutants. As control, cells were cotransfected with these Ran plasmids and the 5GFP plasmid, instead of the 5GFP–maspinNLS plasmid. RanQ69L cannot undergo hydrolysis and therefore it is locked in the GTP bound form, whereas RanT24N has low affinity for GTP and therefore stays always in the GDP bound form. Both mutants have been reported to inhibit Ran‐dependent nuclear import 37. 5GFP–maspinNLS was detected in the nucleus when it was cotransfected with WT Ran‐GTPase (Fig. 3A, upper panels), but not when it was cotransfected with RanT24N (Fig. 3A, middle panels) or RanQ69L (Fig. 3A, lower panels). As expected, 5GFP's subcellular localization was not affected when it was cotransfected with wild‐type or Ran mutants (Fig. 3B). Quantification of the nuclear to cytoplasmic fluorescence ratio (*F*_n_/*F*_c_) in these cells showed that Ran mutants significantly inhibited 5GFP–maspinNLS translocation to the nucleus (Fig. 3C). This result indicates that maspinNLS depends on a functional Ran‐GTPase in order to drive nuclear translocation of the 5GFP construct.

## Discussion

Our results indicate that maspin NLS is able to transport the chimera 5GFP–maspinNLS into the nucleus. However, maspinFL–5GFP does not enter the nucleus. At this point two essential questions need to be addressed – why doesn't maspinNLS promote nuclear translocation of maspinFL? Which importins are involved in maspinNLS‐mediated nuclear translocation? As cnls mapper was primarily designed to identify an importin‐α‐dependent classical NLS 23, we first asked if the predicted NLS would interact with importin α1 (KPNA2), which is considered a general importer of cargoes bearing classical NLS 38. Co‐immunoprecipitation assays and isothermal titration calorimetry, however, did not confirm this hypothesis (data not shown). A recent method called SILAC‐Tp allowed the identification of cargoes for different importins 39. In one of these studies, maspin was ranked high among importin‐11 cargoes and possibly as a transportin‐1 (Kapβ2) cargo as well 40. Interestingly, maspinNLS peptide is suitable for a PY‐NLS signal (KLIKRLYVDKSLNLSTEFISSTKRPYA), which is recognized by transportin‐1 41. We are currently investigating this possibility.

**Figure 4:**
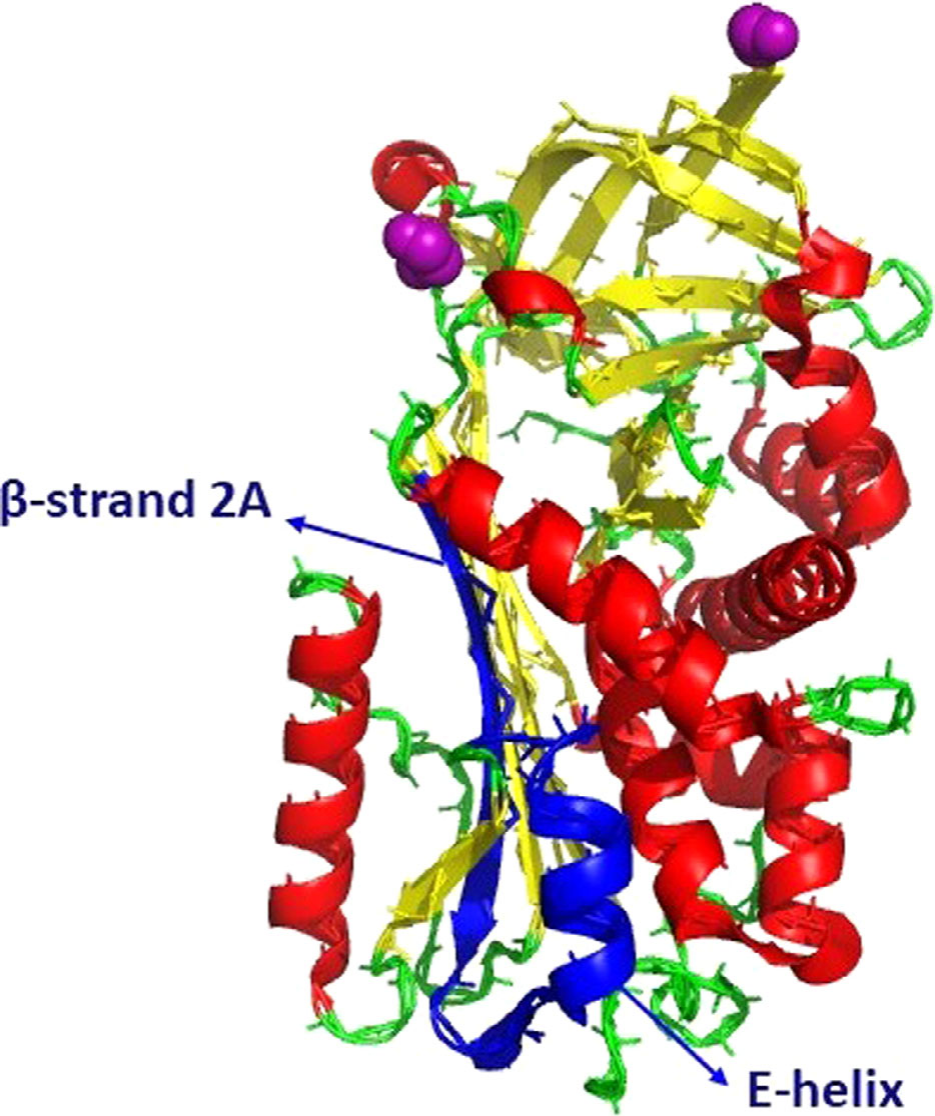
Maspin X‐ray crystal structure 46. Ribbon diagram; α‐helices are in red, β‐strands in yellow, the reactive center loop in purple and coils and turns are in green. The predicted NLS in maspin is shown in blue. Figure was generated with pymol 47. PDB 1WZ9.A.

We currently do not understand why maspinNLS was not able to drive maspinFL nuclear translocation, but we speculate it might be related to the localization of this peptide in maspin's three‐dimensional structure, as part of the maspinNLS (the β‐strand 2A of the β‐sheet A) is buried inside the molecule (Fig. 4). Furthermore, maspin is subjected to several post‐translational modifications, including phosphorylation 22, 42, nitrosylation 43 and acetylation 44. We have previously observed that EGF‐induced maspin phosphorylation is followed by its nuclear translocation 22. As phosphorylation is recognized as an important regulator of nuclear translocation 45, our results support a model in which maspin's NLS is somehow hidden from the nuclear transport machinery (mainly importins). Upon different stimuli (for example, member of the EGF growth factor family), maspin phosphorylation leads to a conformational modification, which may allow NLS exposure. In addition, we observed that maspin can potentially translocate passively to the nucleus, suggesting the presence of different pools of maspin in the cell, which are differentially regulated. In conclusion, our data shed light on the mechanism of maspin nuclear translocation, which may lead to a better understanding of maspin's biological and tumor suppression function.

## Conflict of interest

The authors declare no conflict of interest.

## Author contributions

JR performed experiments and interpreted data. LZ performed experiments. MRMF provided new tools and made manuscript revisions. NP designed experiments, analysed data, supervised the study and made manuscript revisions. NC conceived the study, analysed data, supervised the study and wrote the manuscript.
